# The role of Pressurized IntraPeritoneal Aerosol Chemotherapy in the management of gastric cancer: A systematic review

**DOI:** 10.1515/pp-2018-0127

**Published:** 2019-03-05

**Authors:** Pankaj Kumar Garg, Maximilian Jara, Miguel Alberto, Beate Rau

**Affiliations:** Department of Surgical Oncology, All India Institute of Medical Sciences, Rishikesh, Uttarakhand, India; Department of General Surgery, Campus Virchow Klinikum, Charité, Universitätsmedizin Berlin, Berlin, Germany

**Keywords:** gastric cancer, peritoneal carcinomatosis, Pressurized IntraPeritoneal Aerosol Chemotherapy

## Abstract

**Background:**

The quest to cure or to contain the disease in cancer patients leads to new strategies and techniques being added to the armamentarium of oncologists. Pressurized IntraPeritoneal Aerosol Chemotherapy (PIPAC) is a recently described surgical technique which is being evaluated at many centers for the management of peritoneal metastasis (PM). The present study is a systematic review to evaluate the current role of PIPAC in the management of gastric cancer associated PM.

**Methods:**

A systematic search was conducted in Pubmed and EMBASE database using relevant keywords and confirming to the PRISMA guidelines to identify the articles describing the role of PIPAC in gastric cancer associated PM. All the studies which were published prior to July 1, 2018 in English literature and reported the role of PIPAC in gastric cancer associated PM were included in the systematic review.

**Results:**

The search yielded 79 articles; there were ten published studies which have reported the use of PIPAC in gastric cancer associated PM. A total of 129 patients with gastric cancer associated PM were treated in the studies. Only two studies had an exclusive cohort of gastric cancer patients while eight other studies had a heterogeneous population with a small proportion of gastric cancer patients. There was only one study highlighting the role of PIPAC in neoadjuvant setting to downgrade the peritoneal carcinomatosis index. All the studies revealed that PIPAC is feasible and has minimal perioperative morbidity, even after repeated applications.

**Conclusion:**

There is a scarcity of English literature related to the role of PIPAC in gastric cancer associated PM. PIPAC is a safe and well-tolerated procedure which has the potential to contain spreading PM. Further studies are warranted to better define the role of PIPAC in gastric cancer associated PM.

## Introduction

Gastric cancer is the fifth most common cancer and the second most common cause of cancer-related death worldwide [1]. The median survival of the patients with gastric cancer associated peritoneal carcinomatosis (PM) remains poor. Even systemic chemotherapy has not been able to provide significant survival benefit to these patients. A cochrane review concluded that systemic chemotherapy prolongs overall survival (OS) by approximately 6.7 months more than best supportive care (BSC) (hazard ratio 0.3, 95 % CI 0.24–0.55). Moreover, a combination chemotherapy adds another one month to the OS (HR 0.84, 95 % CI 0.79–0.89) compared to single agent therapy, which is partly counterbalanced by increased toxicity, though it largely gets offset by the drug-induced adverse events [1]. These patients with gastric cancer associated PM are also likely to have a poor and deteriorating quality of life (QOL) due to associated troublesome pain, ascites, bowel obstruction and fistulae.

PM in any solid cancer represents a disseminated disease and is associated with a dismal prognosis. Various guidelines recommend systemic chemotherapy as a therapeutic option for PM in a well-preserved patient and BSC is the norm in a terminally ill patient [2]. Cytoreductive surgery (CRS) and hyperthermic intraperitoneal chemotherapy (HIPEC) has generated a considerable interest in the last three decades in the management of PM. Various studies have suggested that CRS/HIPEC may even prove to be a curative treatment in a select group of patients who have isolated low volume peritoneal disease [3]. A retrospective French study of 277 patients with GC related PM indicated a significant survival benefit with CRS/HIPEC compared to CRS alone (median survival – 18.8 vs. 12.1 months) [4]. A recent prospective study of 35 patients with GC related PM (PCI<6) also highlighted a notable median survival of 19 months when they were treated with CRS/HIPEC [5]. Furthermore, a number of phase III randomized controlled trials (RCTs) are being conducted presently across the world in many centers to better identify the selection criteria for CRS/HIPEC [6]. The strict criteria for selecting the patients for CRS/HIPEC is definitely warranted in order to avoid the associated postoperative morbidity and mortality in those patients in whom this procedure will be futile oncologically and will not add to either progression free survival (PFS) or OS [7]. However, these strict criteria leave a large room for a significant number of patients who are not fit for CRS/HIPEC in view of high-volume peritoneal disease where CRS/HIPEC is likely to leave a significant gross residual disease.

Pressurized IntraPeritoneal Aerosol Chemotherapy (PIPAC), a recently described new surgical technique to administer chemotherapy directly to the peritoneum under pressure, has added a new dimension to the armamentarium of the oncologists to address the PM in those patients who are not suitable candidates for CRS/HIPEC [8]. The first report of successful application of PIPAC in three patients with PM, including one with gastric cancer, was published in 2014 [9]. Since then, a number of articles have described the effectiveness and the safety of PIPAC in PM in patients with cancers of various origins.

## Methods

**Figure 1:**
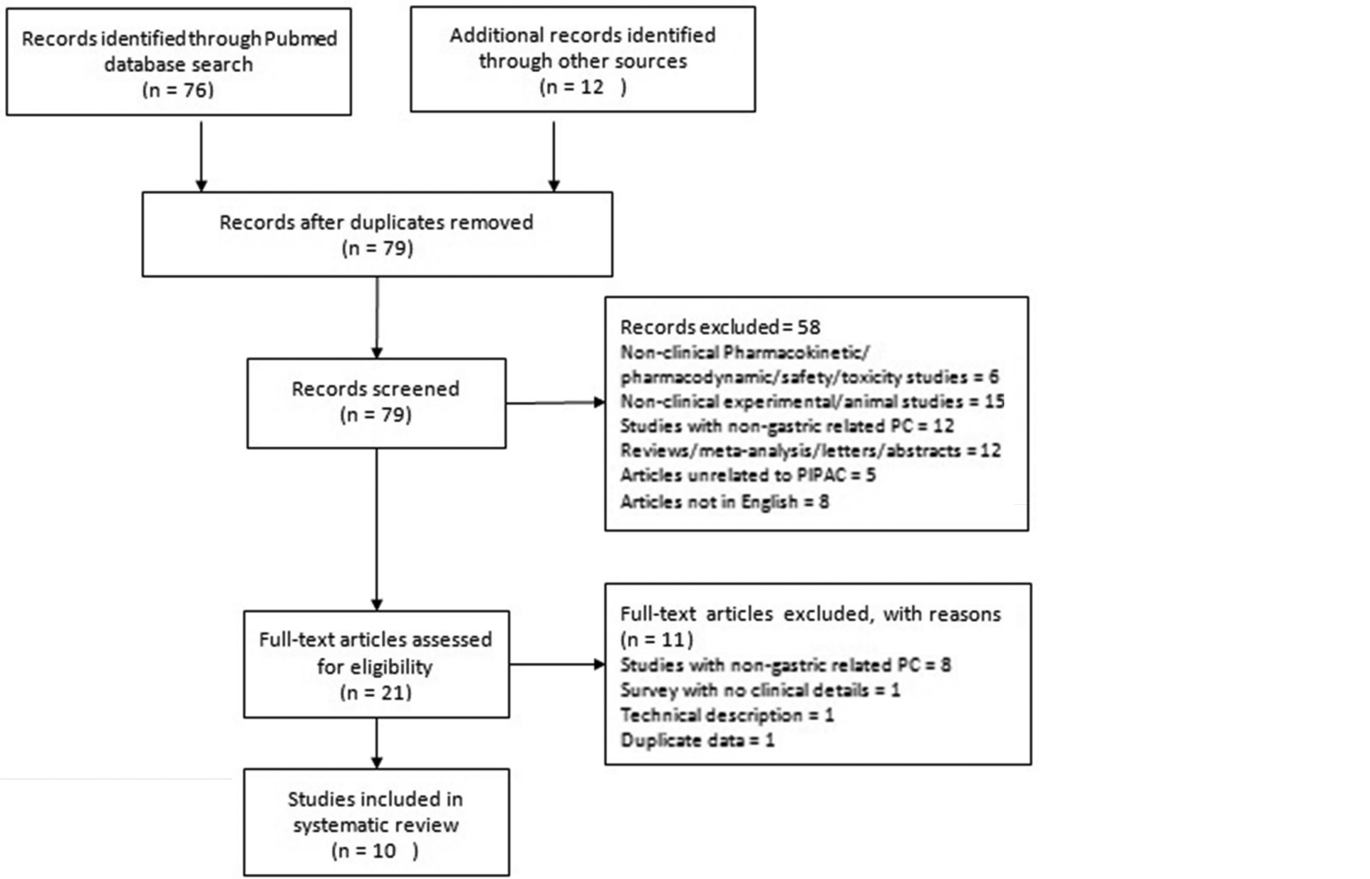
PRISMA flow diagram for the present systematic review.

A systematic search was conducted in Pubmed and EMBASE database using keywords PIPAC[All Fields] OR (Pressurized[All Fields] AND intraperitoneal[All Fields] AND (“aerosols”[MeSH Terms] OR “aerosols”[All Fields] OR “aerosol”[All Fields]) AND (“drug therapy”[Subheading] OR (“drug”[All Fields] AND “therapy”[All Fields]) OR “drug therapy”[All Fields] OR “chemotherapy”[All Fields] OR “drug therapy”[MeSH Terms] OR (“drug”[All Fields] AND “therapy”[All Fields]) OR “chemotherapy”[All Fields])) on July 6, 2018. Inclusion criteria were clinical studies reporting the role of PIPAC in gastric cancer associated PM and published in English language prior to July 1, 2018. Exclusion criteria were (a) systematic reviews/meta-analysis/letters/corrections, (b) non-clinical experimental/animal studies, (c) pharmacodynamic/pharmacokinetic/safety studies without clinical details as Figure 1 shows.

## Results

Ten studies were identified based on inclusion/exclusion criteria, published prior to July 1st, 2018 in English literature, which have reported the use of PIPAC in gastric cancer associated PM [10, 11, 12, 13, 14, 15, 16, 17, 18]. A total of 129 patients with gastric cancer were treated in these studies. There were two studies [10, 11] having an exclusive cohort of gastric cancer patients while eight other studies had a small proportion of gastric cancer patients in their reported cohort of PIPAC. One study highlighted the role of PIPAC in neoadjuvant setting to downgrade the peritoneal carcinomatosis index (PCI) [12]. All but one study reported the use of cisplatin and doxorubicin as preferred chemotherapeutic drugs for PIPAC in patients with gastric cancer. Teixeira Farinha et al. [13] reported oxaliplatin to be used for PIPAC in PM related to colorectal, gastric, small bowel cancer, and pseudomyxoma. PIPAC related adverse events>2 CTCAE (Common Terminology Criteria for Adverse Events) grades, varied from 0 % to 37.5 % in various studies [9, 10, 11, 14, 15, 16, 17]. Six of the ten studies reported QOL data and confirmed that it stabilized or did not deteriorate QOL in the patients who underwent repeated PIPAC procedures [10, 12, 13, 14, 15, 16, 17]. The median survival in the two studies with exclusive cohort of gastric cancer patients was reported to be 13 [11] and 15.4 [10] months. Table 1 highlights the relevant findings of the included studies in the systematic review [9, 10, 11, 12, 13, 14, 15, 16, 17, 19, 20].

**Table 1:** Relevant characteristics reported in include studies.

S.No.	Study	Year	Location	Total patients with PM (n)	Patients with GC associated PM (n)	Intent of PIPAC	Chemotherapy^a^	Average PIPAC procedures per patient	Adverse effect>2 CTCAE	QOL following PIPAC	Remark
1	Graversen et al.	2018	Denmark	35	5	Palliative	Cisplatin and Doxorubicin	3.6^b^	14.2%^b^	Stable	Safe and feasible, associated with histological and cytological regression
2	Teixeira Farinha et al.	2018	Switzerland	42	3	Palliative	Oxaliplatin	2^b^	NR	Stable	No significant systemic toxicity even after repeated PIPAC
3	Alyami et al.	2017	France	73	26	Palliative	Cisplatin and Doxorubicin	2.2^b^	9.7%^b^	NR	PIPAC is feasible along with systemic chemotherapy.
4	Robella et al.	2016	Italy	14	6	Palliative	Cisplatin and Doxorubicin	2.8	0 %	No deterioration	No significant hepatic or renal toxicity.
5	Rezniczek et al.	2016	Germany	63	1	Palliative	Cisplatin and Doxorubicin	NR	NR	NR	Measuring gene expression changes after PIPAC has a predictive and prognostic role.
6	Girshally et al.	2016	Germany	21	3	Neoadjuvant	Cisplatin and Doxorubicin	NR	NR	Stable	PIPAC as an effective neoadjuvant strategy to lower the PCI for good CRS and HIPEC
7	Khomyakov et al.	2016	Russia	31	31	Palliative	Cisplatin and Doxorubicin	1.8	3.2 %	NR	Well tolerated procedure, can induce objective tumor regression
8	Odendahl et al.	2015	Germany	91	29	Palliative	Cisplatin and Doxorubicin	1.7^b^	7.5%^b^	Stable	Potential to stabilize QOL in patients
9	Nadiradze et al.	2015	Germany	24	24	Palliative	Cisplatin and Doxorubicin	2.5	37.5 %	NR	Low-dose PIPAC is safe and associated with objective tumor regression.
10	Solass et al.	2014	Germany	3	1	Palliative	Cisplatin and Doxorubicin	2	0 %	Stable	Complete microscopic peritoneal disease response.

a In patients with gastric cancer.

b For the whole cohort and not exclusively to the patients with gastric cancer.

## Discussion

The two significant limitations of the intraperitoneal chemotherapy are poor penetration and uniform distribution of the drug over the peritoneum. In 2012, a laparoscopy spray of aerosolized drug under-pressure was reported to address these two problems [21]. The authors sprayed methylene blue intra-abdominally using a spraying device consisting of an injector, a line, and a nozzle, inserted through one of the laparoscopic cannula under a capnoperitoneum of 12 mg of Hg for 30 min. The authors reported that there was a uniform staining of the peritoneum, and more so, even the outer aspect of the peritoneum was found to be stained indicating penetration of the dye under pressure. The first clinical experience of PIPAC in PM was published in 2014 [9]. The authors reported their experience of employing PIPAC in three patients of end-stage advanced PM of different origin (gastric, appendiceal, and ovarian each). The procedure was well-tolerated by the patients with no serious adverse effects noted (absence of any>2 CTCAE adverse events). All the patients responded with a decline in PCI score. Moreover, the authors reported histological regression in the peritoneal metastatic tumors; the patient with gastric cancer showed complete histological resolution of the tumor cells. Following this, many articles were published to confirm the safety, feasibility, and effectiveness of the PIPAC. However, most published studies included a heterogeneous population with PM of different origins and patients with different clinic-pathological parameters.

Nadiradze et al. [10] published a study to share their experience of PIPAC in an exclusive cohort of 25 patients with gastric cancer associated PM. One patient could not have even one PIPAC procedure due to inability to access peritoneal cavity in view of extensive peritoneal adhesions. Out of 60 PIPAC procedures in 24 patients (with a mean PCI of 16), the authors reported that 50 % of the patients (12/24) had an objective tumor response with an impressive median survival of 15.4 months. Repeat peritoneal biopsy after PIPAC did not show any visible tumor cell in six patients (complete pathological response). However, the authors admitted that these impressive results should be viewed with caution as a repeat PIPAC was done in those patients who had an objective clinical response (selection bias). Seven patients (7/24) did not have second PIPAC procedure due to progressive disease. The study had a high number of postoperative adverse events>2 CTCAE (9/24, 37.5 %); this can, however, be explained due to inclusion of high risk patients – with extra-abdominal metastasis, with a poor ECOG sore of ≥3, very high PCI, gross ascites, and bowel obstruction.

Another phase 2 prospective trial [11] with an exclusive cohort of gastric cancer patients also reported that PIPAC leads to pathological response (including complete and partial) in almost one third of the patients (60 %) and a median survival of 13 months. It must be noted here that pathological response could be assessed in 15 of the 31 patients who were enrolled in the study and could have had more than one PIPAC procedure.

There have been large variations in the reported adverse events in various studies varying from 0 % to 37.5 %. However, this is largely due to inclusion of a heterogeneous population of patients with PM of different origins and with varying risk factors. Most of the studies involved a few patients of gastric cancer related PM and did not report procedure related adverse events. Alyami et al. [14] reported grades 3 and 4 CTCAE adverse events in 9.7 % and a mortality rate of 6.8 % in their patient cohort. Wound infection, wound dehiscence, and intestinal obstruction were commonly seen adverse event. The common causes of mortality were reported to be progressive disease, aspiration pneumonia, and intestinal obstruction.

The feasibility of PIPAC has been described differently in various studies. Graversen et al. [17] defined PIPAC procedure to be feasible if – (a) a laparoscopic access was possible in 80 % of the patients, (b) the procedure could be completed successfully in 80 % of the patients without any CTCAE grade 4 or 5 events, and (c) if 80 % of the patients could be discharged within 2 days of PIPAC procedure. They reported that PIPAC was feasible for all the patients (35 patients, 129 PIPAC procedures). Nadiradze et al. [10] reported that mere 3 out of the 24 patients could not undergo repeated PIPAC due to non-access to the abdominal cavity in view of severe adhesions.

Whenever any intervention is performed with a palliative intent in any patient with an advanced cancer, it must not deteriorate the current QOL. Even a stabilization of QOL in a terminally ill patient can be considered as a success of a palliative procedure. Six of the nine studies confirmed that repeated PIPAC helps stabilize the QOL and prevent its further deterioration (Table 1).

The next natural question is what the indications of PIPAC are? Almost all studies except one performed PIPAC in patients with high PCI or co-morbid conditions which made them unsuitable for CRS/HIPEC. Obviously, PIPAC in this setting can be regarded as a palliative procedure to improve the quality of life, or at least being able to stabilize it from further deterioration. The ‘one shoe fits all’ approach is not possible in medicine and so, it is of utmost importance to identify patients suitable for a particular procedure to have optimum results. In the time line of the disease progression, a window needs to be identified for PIPAC to be brought in, when the patient does not respond to systemic chemotherapy but still has a reasonable performance status and not having gross ascites, bowel obstruction, or extra-abdominal disease [10].

Can one expect the PCI to drop to that level with repeated PIPAC procedures where one can think of performing secondary CRS/HIPEC; or in other words, can PIPAC be used as a neoadjuvant procedure in patients with high PCI? Girshally et al. reported that neoadjuvant PIPAC is feasible and has the potential to consider secondary CRS/HIPEC in a select group of patients with diffuse small bowel involvement to reduce the extent of CRS [12]. In their patient cohort, twelve of 21 patients had a low PCI (mean 5.8±5.6) and the remaining nine patients were having an advanced peritoneal disease (mean PCI 14.3±5.3) at initial laparoscopy. Repeated PIPAC (3–4 cycles per patient) led to radiological tumor regression in 7/9 patients while major histological regression was made out in 8/9 patients. Though, there were only three patients with gastric cancer in their cohort of 21 patients and none was in the cohort of advanced PM, this study suggests expanding indications of PIPAC in PM.

The present review highlights the paucity of the data related to the role of the PIPAC in gastric cancer related PM. The PIPAC procedure is still in its infancy. Most of the studies had a heterogeneous cohort of the patients of PM of different origins making it difficult to evaluate the true benefit of the procedure in a specific condition. Furthermore, none of the published study has reported the survival benefit of PIPAC in gastric cancer.

As all the studies conducted so far have, at least, established the safety, feasibility, and potential to stabilize the QOL, multiple trials are currently being undertaken at various centers to evaluate the role of PIPAC in gastric cancer associated PM (Table 2). However, one may note that only two out of five trials have exclusive cohort of gastric cancer patients, other trials have a largely heterogeneous population of patients with PM of varying origin.

**Table 2:** Various ongoing trials to evaluate the role of PIPAC in eritoneal carcinomatosis of various origins including gastric cancer (www.clinicaltrials.gov.in as accessed on 12-07-2018).

S.No.	Study title	Peritoneal origin of peritoneal carcinomatosis	Location	Study to be completed
1	IntraPeritoneal Aerosol Chemotherapy in Gastric Cancer	Gastric cancer	Ruhr University of Bochum, Herne, North Rhine Westphalia, Germany	Completed, results not yet published
2	Study of Efficacy and Safety of Laparoscopic Intraabdominal Chemotherapy (PIPAC) Performed in Patients With Peritoneal Carcinomatosis From Colorectal, Ovarian, Gastric Cancer and Primary Peritoneal Tumors	Colorectal, ovarian, gastric, and primary peritoneal tumors	FPO-IRCCS Institute for Cancer Research and Treatment, Candiolo, Turin, Italy	October, 2018
3	PIPAC Nab-pac for Stomach, Pancreas, Breast and Ovarian Cancer	Stomach, pancreas, breast, and ovarian cancer	UZ Ghent, Ghent, East-Flanders, Belgium	December, 2020
4	International Registry of Patients Treated With Pressurized IntraPeritoneal Aerosol Chemotherapy (PIPAC)	Colorectal, ovarian, gastric, appendical, pancreatic, gallbladder, small bowel, pseudomyxoma peritonei, and malignant mesothelioma	Multicentric study	May, 2019
5	Pressurized IntraPeritoneal Aerosol Chemotherapy (PIPAC) With Oxaliplatin In Patients With Peritoneal Carcinomatosis	Gastric cancer	National University Hospital Singapore, Singapore	January, 2019

## Conclusion

The present systematic review clearly highlights the scarcity of English literature to support the role of PIPAC in gastric cancer associated PM. PIPAC is a safe and well-tolerated procedure which has the potential to contain spreading PM. Large studies with an exclusive cohort of gastric cancers are warranted to better define the role of PIPAC in gastric cancer associated PM.
